# Using Quality Improvement to Design and Evaluate an Outpatient Day Treatment Pathway for Pediatric Patients with Diabetes Mellitus Requiring Insulin Initiation

**DOI:** 10.1097/pq9.0000000000000776

**Published:** 2024-11-20

**Authors:** Svetlana Azova, Charumathi Baskaran, Sara Einis, Jennifer Fortin, Marisa Silva, Miriam Gorman, Benjamin Ethier, Sonal Nanavati, Olivia Sterns, Katharine Garvey, Erinn T. Rhodes

**Affiliations:** * From the Division of Endocrinology, Boston Children’s Hospital, Boston, Mass.; † Department of Pediatrics, Harvard Medical School, Boston, Mass.; ‡ Department of Nursing, Boston Children’s Hospital, Boston, Mass.; § Department of Pediatrics, Boston Children’s Hospital, Boston, Mass.; ¶ Department of Population Health, Boston Medical Center, Boston, Mass.

## Abstract

**Introduction:**

Education and management of children with new-onset or established diabetes mellitus (DM) requiring insulin initiation do not always require hospitalization. We developed a pathway for outpatient day treatment of select patients after initial evaluation in the emergency department (ED) at a pediatric, tertiary care academic medical center.

**Methods:**

A multidisciplinary team identified key initial eligibility criteria for outpatient day treatment for insulin initiation, including absence of diabetic ketoacidosis, age ≥3 years, and plasma beta-hydroxybutyrate (BOHB) <1 mmol/L. Electronic medical record reviews and surveys administered to endocrine providers determined exclusions or reasons for nonparticipation. Refinement of the pathway occurred through iterative plan-do-study-act cycles. Statistical process control evaluated the uptake among eligible patients.

**Results:**

We launched the pathway in September 2020. Of 534 patients presenting to the ED with new-onset or established DM requiring insulin initiation in the first 2.5 years, 198 were potentially eligible for day treatment. Of these, 65 children (33%) completed the pathway. One additional patient was hospitalized following Day 1 of education due to newly identified psychosocial stressors. The increase of BOHB cutoff to 1.5 mmol/L and the option of rapid-acting insulin bolus for borderline BOHB resulted in a significant shift in utilization from a mean of 24.4% to 41.1%. Persistent barriers to participation include limited appointment availability, weekend presentation, and patient/family concerns.

**Conclusions:**

Outpatient day treatment was successful for select pediatric patients with new-onset or established DM requiring insulin initiation. However, this approach necessitates flexible resources and supportive patient messaging.

## INTRODUCTION

Pediatric patients with new-onset diabetes mellitus (NODM) or established diabetes mellitus (DM) requiring insulin initiation have traditionally required inpatient admission for ≥1 day for management and education. However, multiple studies indicate that the long-term glycemic control for patients educated in the outpatient or home-based setting is not different from those who received inpatient teaching.^1–3^ Similarly, patients with NODM managed in the outpatient setting had no more frequent acute complications such as diabetic ketoacidosis (DKA) or severe hypoglycemia compared with inpatient treatment groups.^1,2,4^ Parental and patient diabetes knowledge were similar regardless of the education setting.^2^ Several centers worldwide have demonstrated significant cost advantages with the outpatient and home-based NODM care models.^5–7^ Taken together, these data suggest that outpatient NODM management is safe, productive, and cost-effective^8,9^ and that families prefer it.^10^ Given these advantages, many diabetes centers in the United States have moved to an outpatient care model for DM patients requiring insulin initiation. However, this may be logistically challenging for large programs that do not have outpatient space or resources to accommodate this intermittent but intensive education.

In 2012, the inpatient diabetes program at Boston Children’s Hospital (BCH) launched an intensive, multidisciplinary Diabetes Day Treatment Program (DDTP) for NODM to shorten hospital length of stay. This hybrid program admits patients to the inpatient unit for an initial 23-hour observation admission to ensure medical stability and program eligibility. This period is followed by 1–2 consecutive teaching days, lasting 6-8 hours each visit, in BCH’s outpatient Infusion and Day Treatment Center. Quality improvement (QI) efforts established the safety and feasibility of this program within the first few years of its launch. However, as competing demands for inpatient resources have grown, we identified a need for an additional pathway without utilizing an inpatient bed. Discussions about such an alternative began in 2018. However, the onset of the COVID-19 pandemic and associated hospitalizations,^11^ which further exacerbated the problem of limited inpatient bed availability, expedited the process.

We implemented an outpatient day treatment pathway for children presenting to the emergency department (ED) with new or established DM requiring insulin initiation to address this need. We implemented this pathway to bypass an inpatient admission and thus allow for better allocation of inpatient hospital resources. In this new program, the DDTP-ED Referral Pathway launched as a pilot in September 2020, patients receive an initial evaluation and administration of long-acting insulin in the BCH ED, followed by an average of 2 days of teaching in the day treatment setting. We conducted the QI initiative described in this report to design and evaluate the feasibility and safety of this outpatient day treatment program. The initial SMART aim was to increase the proportion of eligible patients with new-onset or established DM who participate in the new DDTP-ED Referral Pathway pilot to 25% by the end of the calendar year 2020.

## METHODS

### Context

BCH is a pediatric, tertiary care academic medical center in Boston, Mass., offering inpatient and outpatient DM care. The hospital has approximately 475 beds, including a medical-surgical intensive care unit and an intermediate care intensive care unit. There are about 25,000 inpatient admissions and approximately 60,000 ED visits per year. The Diabetes Program at BCH manages about 250 patients with NODM annually and has a population of approximately 2200 outpatients with DM. The Division of Endocrinology has 44 physicians, 9 fellows, and 13 outpatient diabetes nurse educators. The inpatient diabetes program, primarily supported through the Department of Nursing Medicine Patient Services, has a full-time inpatient program coordinator, full-time inpatient nurse practitioner, and several inpatient diabetes nurse educators (providing 2–3 full-time equivalent coverage). All inpatient diabetes program staff support and educate patients in the day treatment program. The care team also has support from nutrition and social work providers in inpatient and outpatient settings.

The Infusion and Day Treatment Center at BCH, where the day treatment program takes place, is an outpatient unit and an alternative to inpatient admission for patients requiring infusion therapies and novel medical day treatments. The Center offers patients from infancy to adulthood a place to receive treatment by specialty skilled registered nurses, nurse practitioners, and child-life therapists in a family-centered outpatient setting that provides consistent and ongoing relationships with the healthcare team.

In 2019, an interprofessional team of endocrinologists, diabetes nurses, QI consultants, and representatives from hospital services, including pharmacy, nursing, nutrition, patient financial services, the outpatient Infusion and Day Treatment Center, the ED, and patient care operations, reviewed the existing DDTP. The team identified appropriate eligibility criteria for an entirely outpatient day treatment pathway for patients with new-onset or established DM requiring insulin initiation with entry through the ED. The current criteria are presented in Table 1, representing the summary outcome of the QI efforts to date.

**Table 1.** Eligibility Criteria for the DDTP-ED Referral Pathway

Medical Criteria	Social Criteria
1. Diagnosis of new-onset diabetes (or established diabetes requiring insulin initiation) 2. Not in DKA 3. Plasma BOHB < 1.5 mmol/L. If initial BOHB is 1.5–2.5 mmol/L, administer a rapid-acting insulin bolus and repeat BOHB in 2–3 h to make sure it is < 1.5 mmol/L. 4. COVID-19 symptoms screening and/or test negative	1. Age ≥ 3 y 2. English or Spanish speaking (other languages with advance notice) 3. Family able to contact Endocrine service overnight 4. No significant mental health or cognitive issues 5. Family able to arrive at 8 am for Day 1 of education

### Intervention

**Fig. 1.**
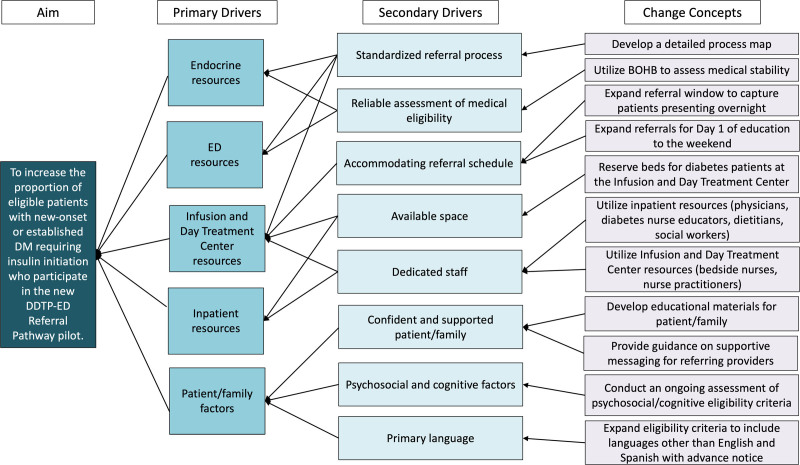
Key driver diagram demonstrating the identified primary and secondary drivers necessary for successfully attaining the aim, with the associated change concepts.

We initially designed the DDTP-ED Referral Pathway pilot to adapt an existing DDTP, which launched in 2012 and provided 1–2 consecutive days of diabetes education through the day treatment program following a 23-hour observation admission. An innovative feature of this model was utilizing the outpatient Infusion and Day Treatment Center setting in combination with inpatient resources and personnel. Utilizing this model and drawing from experiences with the existing DDTP and the best available evidence on quality and safety from the literature, we implemented appropriate modifications in the design of the DDTP-ED Referral Pathway. Our key driver diagram (Fig. 1) depicts our theory for improvement.

We launched the DDTP-ED Referral Pathway pilot in September 2020. Patients presenting to the BCH ED with concern for new or established DM requiring insulin initiation first underwent standard assessment and management, which included ruling out DKA. Criteria for DKA included the presence of plasma glucose >200 mg/dL, acidemia [venous pH < 7.3 (arterial pH < 7.35) or serum total carbon dioxide or venous bicarbonate <18 mmol/L (updated from <15 mmol/L following 2022 guidelines from the International Society of Pediatric and Adolescent Diabetes [ISPAD])], and moderate or large ketonuria or ketonemia (plasma beta-hydroxybutyrate [BOHB] ≥ 3 mmol/L).^12^ Age <3 years has been used as an exclusionary criterion for the existing DDTP pathway and was extended to the DDTP-ED Referral Pathway. We chose this age cutoff due to concern for a higher risk of hypoglycemia following insulin initiation in toddlers, combined with decreased ability to recognize, communicate, and self-manage symptoms of hypoglycemia,^13^ as well as behavioral challenges that may complicate day treatment education and home management within the first few days of diagnosis.

After confirming eligibility for outpatient diabetes management, the patient received a dose of long-acting insulin (eg, insulin glargine) on the lower end of the recommended range in the existing clinical management guidelines for NODM (and patients with established DM who are new to insulin) to decrease the risk of hypoglycemia (s**ee Table 1, Supplemental Digital Content 1,** http://links.lww.com/PQ9/A613).^14^ The ED staff discussed DDTP expectations with the family, and they provided a family education sheet (available in English and Spanish), which included an overview of the DDTP and contact information for the on-call endocrinologist in case of any issues. The patient was then discharged home with a plan to return to BCH’s outpatient Infusion and Day Treatment Center for their Day 1 DDTP appointment. To ensure the availability of resources, the DDTP-ED Referral Pathway pilot limited referrals for Day 1 of education to 1 patient each day, Monday to Friday.

The outpatient education lasts an average of 2 consecutive days. It consists of formal sessions with the endocrinologist(s) (either fellow and attending or diabetes hospitalist), diabetes nurse educators, a dietitian, and a social worker on the inpatient diabetes service at that time. The family also participates in all mealtime diabetes skills under the supervision of a registered nurse and nurse practitioner. The Infusion and Day Treatment Center staff sends all prescriptions during Day 1 of education, and the diabetes nurse educator reviews the use of the home supplies before discharge on the first day. The families receive instruction to call the on-call endocrinologist in the evening following Day 1 to review nighttime skills and ask questions between education days. The patient and family return for Day 2 of education the following day. We include a sample schedule for outpatient education based on a 2-day plan in **Figure 1, Supplemental Digital Content 3**, http://links.lww.com/PQ9/A615).

We refined the pathway after iterative plan-do-study-act (PDSA) cycles. We include a timeline of the PDSA cycles associated with refinements to the DDTP-ED Referral Pathway in **Table 2, Supplemental Digital Content 2,** http://links.lww.com/PQ9/A614. PDSA cycles focused on adjustment of referral times and modification of eligibility criteria. In PDSA cycles 1 and 4, we modified the referral window to capture patients presenting to the ED late in the day. In the first PDSA cycle, we extended the ED arrival time to 9 pm, and patients returned for Day 1 education the following day. For further flexibility, in PDSA cycle 4, patients who presented late in the evening or overnight were allowed to return for Day 1 of education within 24–36 hours of ED presentation as long as they were deemed medically stable. In addition, to ensure that families receive effective outpatient education, we added additional screening criteria for significant mental health or cognitive concerns in caregivers.

In PDSA cycles 2, 3, and 5, we modified the BOHB eligibility criteria based on safety assessments and responses to the intervention. Upon presentation to the BCH ED, ED providers performed an initial evaluation to assess for DKA and to manage hydration status. Plasma BOHB, which typically takes approximately 1 hour to result after collection, determined the level of ketosis. ISPAD recommends a cutoff of ≥3 mmol/L to define DKA.^12^ However, a value of ≥5.3 mmol/L has recently been found to have higher specificity and optimal accuracy (91%) for predicting DKA in children presenting to the ED with hyperglycemia.^15^ For the DDTP-ED Referral Pathway pilot, we used a conservative initial cutoff for eligibility of BOHB <1 mmol/L. We increased this cutoff to <1.5 mmol/L in PDSA cycle 2. In PDSA cycle 3, for patients presenting with an initial BOHB in the borderline range (1.5–2.5 mmol/L), a repeat BOHB of <1.5 mmol/L post-fluid bolus was deemed acceptable for eligibility. In PDSA cycle 5, if the initial BOHB was in the borderline range (1.5–2.5 mmol/L), the ED staff gave a rapid-acting insulin (eg, insulin lispro) bolus based on blood glucose correction factor calculations using previously established dosing guidelines (**Table 1, Supplemental Digital Content 1,** http://links.lww.com/PQ9/A613).^14^ A repeat BOHB after 2–3 hours was considered acceptable for eligibility if <1.5 mmol/L.

### Study of the Intervention

We collected data to identify eligible patients and reasons for exclusion or nonparticipation. Sources for data included automated daily reports from the electronic medical record that included all patients evaluated in the ED for DM, as well as chart review to identify patients who presented with new-onset or established DM requiring insulin initiation. For potentially eligible patients who did not participate in the DDTP-ED Referral Pathway pilot, we sent a REDCap survey^16^ to the referring endocrine provider to determine the reasons for exclusion or nonparticipation. We collected feedback on the process through the survey, a feedback email, and regular stakeholder meetings.

### Measures and Analysis

The primary outcome measure was the percentage of eligible new or established DM patients requiring insulin initiation who completed the DDTP-ED Referral Pathway. To understand the process, we measured the reasons for exclusion and nonparticipation among potentially eligible patients. We considered safety concerns, including severe hypoglycemia during outpatient education and the need for admission from the DDTP, as balancing measures. We used statistical process control to evaluate the primary outcome measure on a p-chart and used established rules to identify special cause variation.^17^ A Pareto chart evaluated the relative impact of different reasons for nonparticipation. This project was undertaken as a QI initiative aimed at improving care at BCH and consistent with the QI project guidelines of the BCH Department of Pediatrics, sanctioned by the institutional QI review policy set by the BCH institutional review board.

## RESULTS

**Fig. 2.**
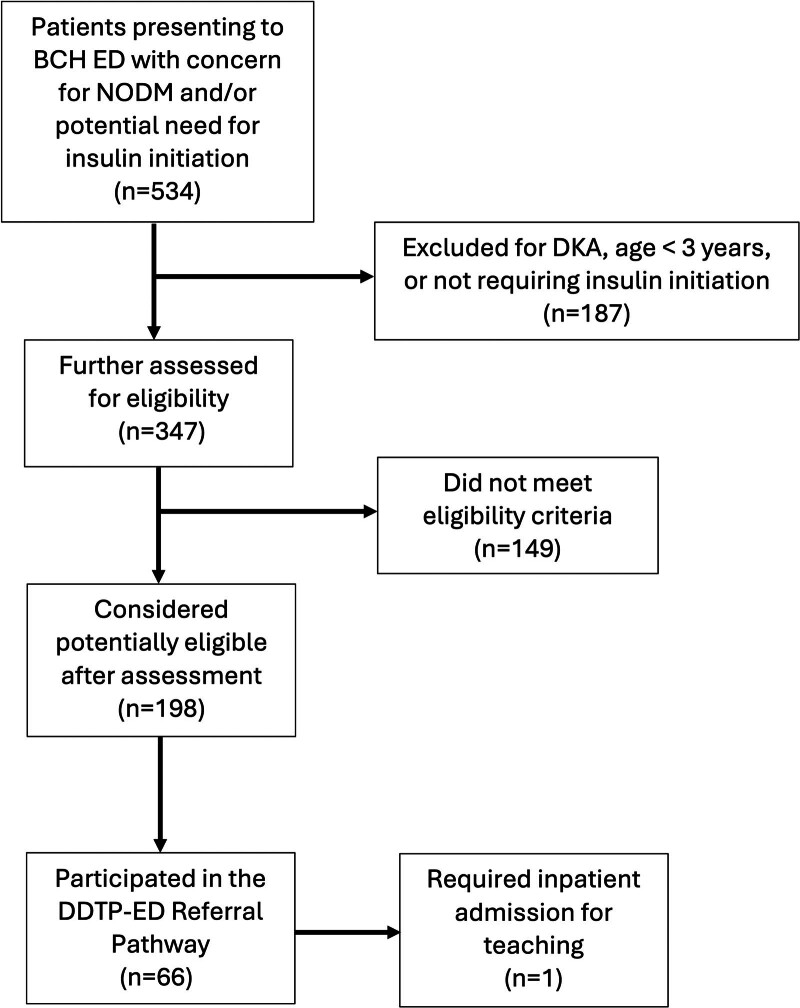
Flowchart demonstrating the utilization of the DDTP-ED Referral Pathway between September 2020 and March 2023.

During the 2.5-year evaluation (September 2020–March 2023), 534 patients presented to the BCH ED with new or established DM that required insulin initiation (Fig. 2). As shown in Figure 2, of the 534 patients, 187 (35%) patients were either in DKA, were <3 years of age, or did not require insulin and were excluded from eligibility for the outpatient day treatment program. Of the remaining 347 patients who were further assessed, 198 (57%) were considered potentially eligible for the DDTP-ED Referral Pathway, and, of these, 66 (34%) participated in the pathway. These patients were 50% male and ranged in age from 3 to 21 years (mean 12.2±4.7 y); they did not differ significantly from potentially eligible patients who did not participate in the pathway in sex distribution or mean age.

**Fig. 3.**
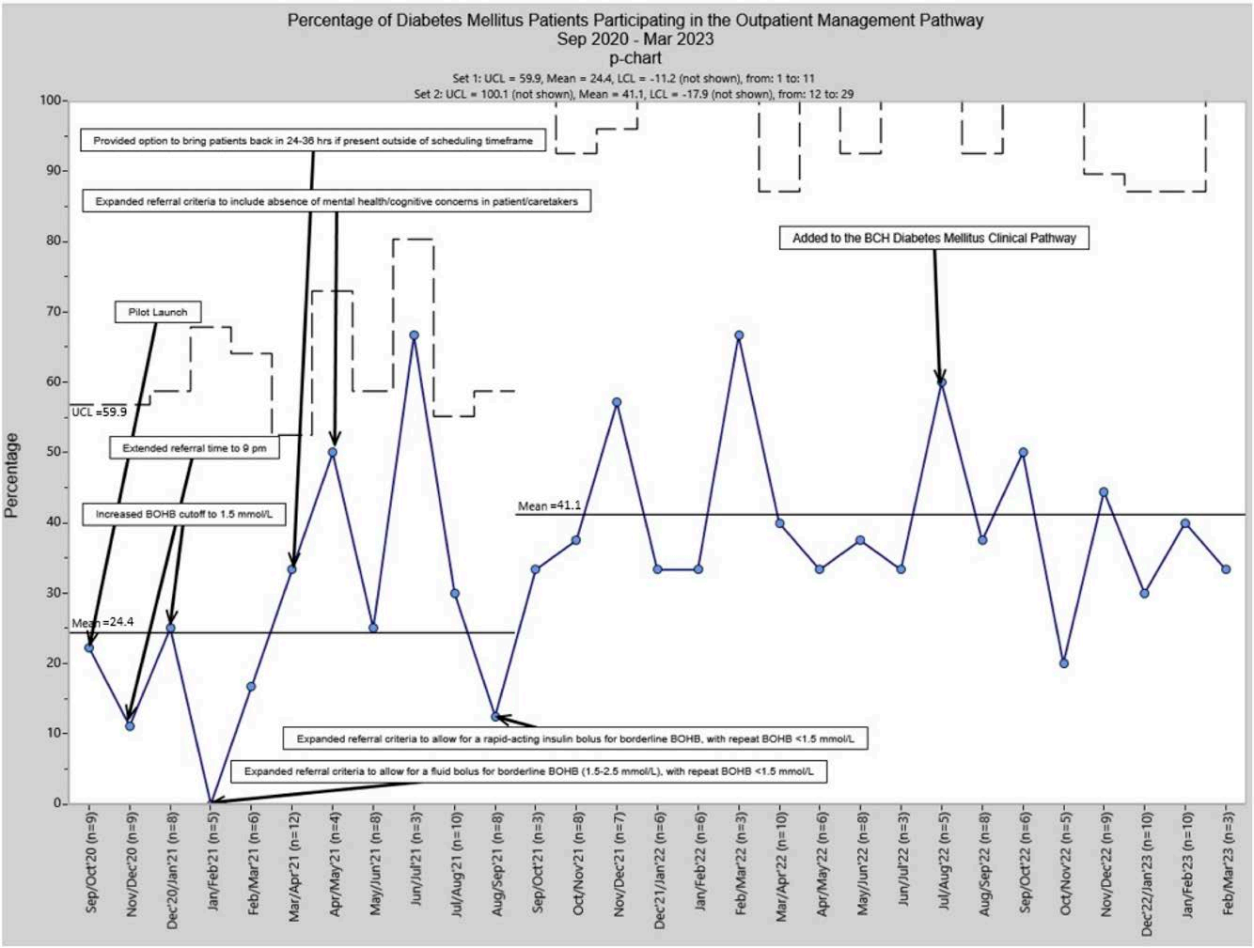
P-chart displaying the percentage of eligible patients participating in the DDTP-ED Referral Pathway between September 2020 and March 2023. LCL, lower control limit; UCL, upper control limit.

**Fig. 4.**
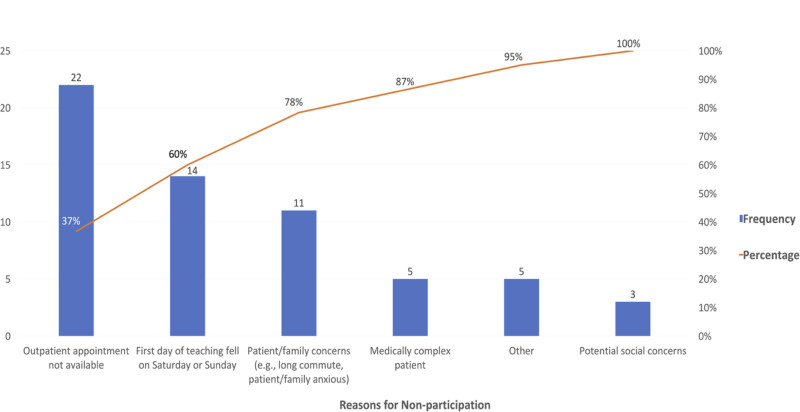
Pareto chart of reasons for nonparticipation in the DDTP-ED Referral Pathway between September 2021 and March 2023.

Using a control chart, we tracked DDTP-ED Referral Pathway completion among eligible patients monthly between September 2020 and March 2023 (Fig. 3). There was a significant shift in the mean from 24.4% before September 2021 to 41.1% beginning in September 2021, associated with PDSA cycle 5. This cycle incorporated the introduction of a rapid-acting insulin bolus for borderline initial BOHB 1.5–2.5 mmol/L, with repeat BOHB <1.5 mmol/L establishing eligibility. Endocrine provider surveys (total response rate 86%) identified reasons for nonparticipation in the DDTP-ED Referral Pathway. We plotted these on a Pareto chart. Since September 2021, coinciding with the last PDSA cycle, the most commonly identified barriers, which accounted for 78% of all reasons for nonparticipation, included lack of appointment availability in the Infusion and Day Treatment Center, weekend presentation (associated with limited staffing/resources), and patient/family concerns/discomfort (Fig. 4). Long commute times and other medical and social concerns were other reasons for nonparticipation and for which inpatient admission was considered appropriate.

Regarding safety outcomes, only 1 patient required inpatient admission after completing Day 1 of day treatment due to newly identified psychosocial stressors. There were no readmissions following the completion of day treatment through the DDTP-ED Referral Pathway and no episodes of severe hypoglycemia during day treatment. Similarly, there were no readmissions or episodes of severe hypoglycemia among patients who participated in the original DDTP (ie, following a 23-h observation admission) during the same period (September 2020–March 2023). In August 2022, the DDTP-ED Referral Pathway became part of the existing BCH clinical pathway for management of new-onset or established DM.

## DISCUSSION

We successfully implemented a pediatric outpatient day treatment pathway for patients with new-onset or established DM requiring insulin initiation at a pediatric, tertiary care academic medical center, and the results indicate that diabetes education in this format is feasible in select patients without significant ketosis. Refining the pathway using QI methodology led to modifications, including changes to the BOHB cutoff for eligibility, providing a rapid-acting insulin bolus to decrease the BOHB into the goal range, and extending the return time to 24–36 hours. These changes resulted in a significant increase in pathway uptake from 24.4% to 41.1% of eligible patients.

The DDTP-ED Referral Pathway is similar to other programs previously described in the literature that have successfully adopted an outpatient education pathway for NODM.^8^ One innovative aspect of the DDTP-ED Referral Pathway was using plasma BOHB as a marker of metabolic stability in the eligibility assessment. American Diabetes Association and ISPAD recommend blood BOHB measurement to diagnose DKA.^18^ In the DDTP-ED Peferral Pathway pilot, the use of BOHB did not compromise the feasibility of the pathway or the safety of the patients participating in the program. We initially used a conservative cutoff of <1 mmol/L and subsequently increased it to <1.5 mmol/L, a threshold value associated with 100% negative predictive rates for DKA diagnosis.^18,19^ Broadening the eligibility criteria over time, including changing the BOHB cutoff from <1 to <1.5, has similarly not resulted in increased safety concerns but expanded access to participation. These outcomes highlight the importance of robust utilization of QI methodologies in planning, adapting, and evaluating a pilot program, resulting in successful implementation.

Other programs offering outpatient education and management for patients with DM requiring insulin initiation have utilized the patient’s home^2,3,10^ or an ambulatory clinical setting.^1,4–9^ Recognizing the logistical challenges to these designs at BCH, the availability of the outpatient Infusion and Day Treatment Center, combined with the utilization of the inpatient diabetes program resources, provided the ideal setting for the DDTP-ED Referral Pathway pilot. This innovative model had the dual purpose of ensuring that the education received by patients with new-onset or established DM requiring insulin initiation was the same regardless of where it occurred (ie, inpatient or day treatment setting) and simultaneously preserving routine outpatient clinical space and resources (endocrinologists, nurse educators, dietitians, and social workers) to provide reliable, dedicated care to the large population of existing patients with DM, including in time-sensitive situations such as acute illness.^20^ However, lack of appointment availability and patient/family concerns were among the main reasons for nonparticipation in the pilot. Recent efforts to support the program’s sustainability have included extending referral for Day 1 of education to Saturdays beginning in March 2024 and ongoing work to provide supportive patient/family messaging about the efficacy and safety of the DDTP-ED Referral Pathway.

Several limitations should be noted. We conducted this initiative at a single institution, so the results may not be generalizable. In addition, we could only determine the reasons for some patients’ nonparticipation. Finally, because this was a pilot program, space was limited to 1 patient per day of teaching. As this system resource was difficult to modify during this initiative, we focused our improvement efforts on other potentially modifiable elements of the process.

## CONCLUDING SUMMARY

Outpatient day treatment is feasible for select patients with new-onset or established DM requiring insulin initiation. Flexible resources and supportive patient messaging regarding outpatient education are necessary for optimal utilization. Adding the DDTP-ED Referral Pathway pilot program to the existing BCH clinical pathway for management of new-onset or established DM in August 2022 has facilitated a mechanism for sustainability. We also extended the pathway in 2022 to several network hospital EDs and are exploring further extension to primary care pediatrician offices. In the future, opportunities may exist to evaluate clinical outcomes and cost-effectiveness.

## ACKNOWLEDGMENTS

The authors would like to thank Kristie Aamodt, MD, PhD, for her assistance in implementing the initiative and Amy Starmer, MD, MPH, Rebecca Hirsch, MPH, CPHQ, and David Johnson, MS, for their support with this clinical pathway initiative as members of the Department of Pediatrics Quality Program at BCH.

## Supplementary Material


