# Supplementary material for: Using Quality Improvement to Design and Evaluate an Outpatient Day Treatment Pathway for Pediatric Patients with Diabetes Mellitus Requiring Insulin Initiation
Source: Pediatr Qual Saf. 2024 Nov 20;9(6):e776. doi: 10.1097/pq9.0000000000000776 (PMC11578214; doi:10.1097/pq9.0000000000000776)
Supplement: Supplementary file 1 [file pqs-9-e776-s001.pdf]

Title: Using Quality Improvement to Design and Evaluate an Outpatient Day Treatment Pathway for Education and Management of Pediatric Patients with Diabetes Mellitus Requiring Insulin Initiation

First Authors: Svetlana Azova, MD, Charumathi Baskaran, MD

SDC, Table 1. Emergency Department Insulin Dosing Guidelines for the Diabetes Day Treatment Program-Emergency Department Referral Pathway

	TDD (units/kg)	Insulin Glargine (Lantus) Dose (units/kg)	Correction Factor Calculation for Rapid-Acting Insulin Bolus
HbA1c < 7% (any age)	0.15	0.075	1500 / (TDD in units/kg x patient's weight in kg)<br><br>This gives by how much 1 unit of rapid-acting insulin analog is expected to lower the blood glucose concentration to the designated blood glucose target of **150 mg/dL** for all ages.
Age < 6 years (any HbA1c)*	0.15	0.075	
Age ≥ 6 and Pre-pubertal	0.25	0.125	
Pubertal	0.5	0.25	
Post-pubertal	0.25	0.125	

* Young children are more vulnerable to severe hypoglycemia, especially given their inability to communicate symptoms of hypoglycemia. Use clinical judgment when selecting the total daily dose of insulin and insulin glargine (Lantus) dose for these patients.

Note: Rounding recommendations for insulin administration via syringe:

- 0.1-0.3 unit → round down
- 0.4-0.7 unit → round to 0.5 unit
- 0.8-0.9 unit → round up to 1 unit

Abbreviations: HbA1c, hemoglobin A1c; TDD, total daily dose.
