# Supplementary material for: Using Quality Improvement to Design and Evaluate an Outpatient Day Treatment Pathway for Pediatric Patients with Diabetes Mellitus Requiring Insulin Initiation
Source: Pediatr Qual Saf. 2024 Nov 20;9(6):e776. doi: 10.1097/pq9.0000000000000776 (PMC11578214; doi:10.1097/pq9.0000000000000776)
Supplement: Supplementary file 2 [file pqs-9-e776-s002.pdf]

Title: Using Quality Improvement to Design and Evaluate an Outpatient Day Treatment Pathway for Education and Management of Pediatric Patients with Diabetes Mellitus Requiring Insulin Initiation

First Authors: Svetlana Azova, MD, Charumathi Baskaran, MD

SDC, Figure 1. Daily Visit Schedule for the Diabetes Day Treatment Program-Emergency Department Referral Pathway

DAY 1: Introduction, Insulin Basics, and Skills-based Learning

8 am – 4:30 pm (~8 hours)

Goal: Patient/Family demonstrates competency in blood glucose/ketone monitoring and insulin administration prior to discharge.

8:00 am:
- Program Overview with Patient/Family
- RN/NP assess patient, obtain blood glucose/ketones, and page Diabetes Service
- NP sends diabetes prescriptions to CVS

8:45 am: **Breakfast and Skills**

9:45 am:
- Diabetes MD(s) Evaluation Day 1

10:15 am:
- SW Assessment Day 1

11:00 am:
- RD Education Day 1
- Meal Plan for remainder of DDTP (RD and RN)

12:15 pm: **Lunch and Skills**
- Family picks up supplies in CVS

1:30 pm
- DNE Education Day 1
- Supply review and call-in instructions

4:30 pm
- Review plan for return time and expectations for Day 2
- Family option to stay for dinner if want more skills practice

DAY 2: General Management Concepts, Nutrition, Exercise, Sick Day, Glucagon

8 am – 1 pm (~5 hours)

Goals:

A) Patient/Family will be proficient in blood glucose/ketone monitoring and insulin administration.

B) Patient/Family demonstrates understanding of fundamental diabetes management and nutrition concepts.

8 am:
- RN/NP assess patient, obtain blood glucose/ketones, and page Diabetes Service
- **Breakfast and Skills**

9 am:
- RD Education Day 2

10 am:
- DNE Education Day 2

11:45 am:
- Diabetes MD(s) Evaluation Day 2

12 pm
- **Lunch and Skills**
- Evaluate need to return for Day 3

1 pm
- SW Check-in Day 2

1:30pm
- Discharge to Home

Abbreviations: DDTP, Diabetes Day Treatment Program; DNE, diabetes nurse educator; MD, medical doctor; NP, nurse practitioner; RD, registered dietitian; RN, registered nurse; SW, social work.

Figure Legend

A typical example of the daily visit schedule for the Diabetes Day Treatment Program-Emergency Department Referral Pathway.
