# Supplementary material for: Using Quality Improvement to Design and Evaluate an Outpatient Day Treatment Pathway for Pediatric Patients with Diabetes Mellitus Requiring Insulin Initiation
Source: Pediatr Qual Saf. 2024 Nov 20;9(6):e776. doi: 10.1097/pq9.0000000000000776 (PMC11578214; doi:10.1097/pq9.0000000000000776)
Supplement: Supplementary file 3 [file pqs-9-e776-s003.pdf]

Title: Using Quality Improvement to Design and Evaluate an Outpatient Day Treatment Pathway for Education and Management of Pediatric Patients with Diabetes Mellitus Requiring Insulin Initiation

First Authors: Svetlana Azova, MD, Charumathi Baskaran, MD

SDC, Table 2. Timeline of Refinements to the Diabetes Day Treatment Program-Emergency Department Referral Pathway

**PDSA Cycle**	**Date**	**Description**	**Outcome**
Launch	9/28/2020	Initial pathway	
PDSA 1	11/2/2020	Extended referral time from 7:30 pm to 9 pm	Adopt
PDSA 2	12/1/2020	Increased BOHB cutoff from 1 to 1.5 mmol/L	Adopt
PDSA 3	1/29/2021	Expanded referral criteria to include BOHB < 1.5 mmol/L post fluid bolus with initial BOHB 1.5-2.5 mmol/L	Adapt
PDSA 4	4/1/2021	Added option to have patients who present overnight (9 pm-7:30 am), outside of standard referral time frame, to return within 24-36 hours. Expanded referral criteria to include absence of significant mental health or cognitive concerns in patient/caretakers.	Adopt
PDSA 5	9/1/2021	Changed referral criteria to include repeat BOHB < 1.5 mmol/L 2-3 hours post-rapid-acting insulin bolus for initial BOHB 1.5-2.5 mmol/L	Adopt

Abbreviations: BOHB, beta-hydroxybutyrate, PDSA, plan-do-study-act.
